# Association of Stress Management with Success of Smoking Cessation in Korean Female Emotional Labor Workers for Service and Sales

**DOI:** 10.3390/ijerph18063023

**Published:** 2021-03-15

**Authors:** Haena Kim, Kang-Sook Lee

**Affiliations:** 1 Department of Public Health, Graduate School, The Catholic University of Korea, 222 Banpo-daero, Seocho-gu, Seoul 06591, Korea; hn0809k@catholic.ac.kr; 2 Department of Preventive Medicine, College of Medicine, The Catholic University of Korea, 222 Banpo-daero, Seocho-gu, Seoul 06591, Korea; 3 Seoul Tobacco Control Center, 222 Banpo-daero, Seocho-gu, Seoul 06591, Korea

**Keywords:** smoking cessation, counseling, stress management, female emotional labor workers

## Abstract

Emotional labor is paid work that involves managing and regulating one’s emotions during the job including evoking and suppressing one’s feelings. This study examined the factors associated with successful smoking cessation through tailored smoking cessation counseling including stress management among female emotional labor workers. The study was conducted from 1 September 2015 to 31 December 2017. A total of 2674 women registered in the Comprehensive Smoking Cessation Service System and were grouped as either emotional labor workers for service and sales (1002) or other occupations (1672) for analysis. The participants received nine sessions of face-to-face and telephone smoking cessation counseling over 6 months, and follow-up assessments were conducted 4, 6, 12, and 24 weeks after counseling. Smoking cessation counseling involved a stress management program comprising stress tests, depression tests, color therapy, and a buddy program including peer support. Factors associated with successful smoking cessation included the number of counseling sessions, motivation rulers (Importance, Confidence, Readiness), average daily smoking amount, expired carbon monoxide (CO), and nicotine dependence. The most associated factor was the number of counseling sessions. Since counseling focused on stress management, it was the most important factor in smoking cessation, and continuous counseling could help those wanting to quit smoking.

## 1. Introduction

In the 1970s, the Korean economy experienced de-agriculturalization and rapid industrialization, marked by rising proportions of manufacturing and service industries. In the 1990s, rapid de-industrialization occurred, where the proportion of employment in the manufacturing industry declined. The share of employment in the manufacturing sector decreased rapidly from 27.8% in 1989 to 16.8% in 2019, whereas employment in the service industry grew equally rapidly from 45.3% to 69.2% [1]. As the Korean industrial structure has become centered on the service industry, it is estimated that approximately 7 million workers are currently engaged in customer service, representing 35.1% of total wage workers [2]. The labor force participation of Korean women aged 15 years and above has continued to grow, reaching 53.5% in 2019 and representing 12 million economically active people [1]. Female workers in the service industry exceeded the number of male workers in 2005 [3].

Emotional labor is paid work that centrally involves managing and regulating one’s emotions for the job including evoking and suppressing one’s feelings [4,5]. Most of the emotional labor workers are in service and sales jobs. Appropriate expression of emotions as desired by the organization requires individual effort, planning, and control [6], and it has been observed that the intensity of emotional labor of service workers is much higher than that of managerial and white-collar workers and physical laborers [7]. Most female emotional labor workers work for organizations that do not offer them guaranteed jobs (i.e., regular work) and are forced to choose such jobs due to the fact of gaps in their careers and lack of other professional skills. As emotional labor workers work as non-regular and dispatched workers [8], breaks at work are often very short and cannot be used freely due to the poor spaces allocated for breaks. This indicates that female emotional labor workers are socioeconomically vulnerable.

By engaging in emotional labor, workers experience a sense of self-alienation due to the emotional dissonance, with severe cases being associated with absenteeism, substance abuse, and alcohol addiction [4]. Continuous emotional labor leads to negative outcomes such as burnout and emotional exhaustion [9,10]. Further, it has been reported to increase job stress and reduce job satisfaction and is negatively correlated with mental health [11,12]. The psychological stress caused by emotional labor may lead to unhealthy behaviors such as smoking [13,14]. The increasing stigmatization of certain socio-economically disadvantaged and ethnic minority groups who continue to smoke, coupled with the spatial segregation of poor and minority populations, may compound to produce “smoking islands”, reinforcing rather than discouraging continued smoking [15]. The working environment of women engaged in customer service positions, such as department stores, call centers, and discount stores, can be regarded as a type of smoking island. Smoking rates are higher among women who are engaged in service-providing labor entailing higher emotional burnout and lower income [16]. Acute stress has been associated with increased cigarette use and reduced likelihood of quitting smoking, and the correlation between stress and smoking is stronger among women than men [17]. While men are more likely to smoke in social situations, women are more likely to smoke as a psychological coping mechanism against stress, anger, and depression [18]. Women smokers are aware of the disadvantages of smoking, and many of them desire to quit. However, women who smoke to reduce stress face difficulties in quitting due to the presence of concerns about managing stress without cigarettes after they quit smoking [17,19].

Smoking among women increases the risk for various diseases such as early menopause, breast cancer, and cervical cancer. Furthermore, smoking during pregnancy increases the likelihood of miscarriage, premature birth, and low birth weight [20]. Although women generally smoke less than men, their dependence is higher, and they experience more severe withdrawal symptoms [21]. Therefore, unlike men, successful smoking cessation for women requires stress management, social support, and improved confidence and readiness [22]. Successful smoking cessation among women smokers requires specific counseling and programs that are tailored for women [23,24].

Previously, anti-smoking services, such as public health center clinics for smoking cessation and non-smoking phone counseling, were centered on adult men. Korean women tend to hide the fact that they smoke because of the social atmosphere that bans women from smoking. Therefore, the percentage of women using non-smoking services was low. Existing anti-smoking support services were not effective in reducing women’s smoking rate, requiring different services from men to aid smoking cessation attempts and practices. The study’s counseling program was designed to help women smokers practice smoking cessation and increase its success rate.

This study classified service workers, such as telemarketers and sales workers, as emotional labor workers for service and sales. The aim of this study was to examine the factors associated with successful smoking cessation through tailored smoking cession counseling with stress management among women emotional labor workers, a population previously neglected by extant smoking cessation support services.

## 2. Materials and Methods

### 2.1. Research Participants

Data from a total of 2674 participants registered with the Comprehensive Smoking Cessation Service System (nosmk.khealth.or.kr) were analyzed in this study. The data were acquired between 1 September 2015 and 31 December 2017. Participants included female smokers aged 19 years and above, who either lived or worked in Seoul. In particular, this study provided a visiting smoking cessation counseling service for women smokers working in businesses with a high proportion of women such as department stores, supermarkets, and call centers.

This study was approved by the Institutional Review Board of the Catholic University of Korea (MC18EESI0103).

### 2.2. Measurement

#### 2.2.1. General and Smoking-Related Characteristics

Participants’ characteristics included age, body-mass index (BMI), drinking status, age at first smoking experience, daily average number of cigarettes smoked at the time of registration, expired carbon monoxide concentration at the time of registration, nicotine dependence, past smoking cessation attempts, motivation rulers, and the number of smoking cessation sessions. It was collected in a self-filled manner at the time of the initial registration consultation to determine the general characteristics of the participant.

#### 2.2.2. Classification of Occupation

Emotional labor includes labor that requires face-to-face or verbal interaction with consumers, labor involving emotional expression to consumers, and labor in which the employer controls the emotions that the workers are permitted to express [4]. This study classified service workers and sales workers as emotional labor workers for service and sales. Managerial, professional, functional, and other employees were classified as other occupations.

#### 2.2.3. Drinking Status

Based on the World Health Organization criteria [25], high-risk drinking refers to drinking habits that may adversely impact the body due to the intake of alcohol over 60 g per time for men and 40 g for women. This study classified adult women who consumed more than five drinks a day (or three cans of beer) twice or more per week as the high-risk drinking group. Participants who did not drink in the past year were marked as non-drinking, and those who typically had four or fewer drinks per day or once or less than once per week were classified as the appropriate drinking group.

#### 2.2.4. Nicotine Dependence

We used the Fagerstrom Test for Nicotine Dependence (FTND). It is 10 point scale, with higher scores indicating stronger nicotine addiction. Nicotine dependency scores were categorized into low (0 ≤ FTND score ≤ 3), normal (4 ≤ FTND score ≤ 6), and high (7 ≤ FTND score ≤ 10) [26].

#### 2.2.5. Motivation Rulers

The importance attributed by the participants to quitting smoking was assessed using the question, “How important is quitting smoking to you?”, rated from 0 (Not important at all) to 10 (Most important goal of my life). The participants’ confidence about tobacco cessation was assessed with the question, “How confident are you that you will quit smoking within the next month?”, rated on a scale from 0 (Not at all) to 10 (100% Confident). Readiness to quit was assessed using the question, “How ready are you to quit smoking within the next month?”, rated on a scale from 0 (Not at all) to 10 (100% Ready) [27,28].

#### 2.2.6. Supporters

Participants were asked to respond to the question, “Who helps you quit smoking?” by choosing all options that applied from the following: parents/grandparents, siblings, spouse/lover, children, friends (personal/school), coworkers, and others.

#### 2.2.7. Times When the Temptation to Smoke Is the Highest

Participants were asked to respond to the question, “What time of day are you most tempted to smoke?” by choosing all options that applied from the following: as soon as I wake up in the morning, before I go to bed, after a meal, in the restroom/after shower, during breaks, times when I smoke out of habit (when I need energy, when I realize that I had not smoked a cigarette, when I drink alcohol or coffee, and when I am alone or am waiting for someone), in positive situations (when I am with friends or family and when I am having a conversation or trying to ease the day’s fatigue), in negative situations (when I am stressed, when things do not work out as planned, when I get mad, when I am with another smoker, and when I see someone smoking on TV), etc.

#### 2.2.8. Smoking Cessation Counseling for Women

1. Participant recruitment

The participants were recruited through an advertisement showing the hazards of smoking and the benefits of smoking cessation during regional health-related campaigns (World No Tobacco Day, World Oral Health Day, Women’s Day, Seoul Health festival, Health campaigns in local districts, etc.) and sports events for women (Baseball Queens Day, Women’s marathons, etc.). Most of the recruits had other occupations. Counseling was provided at business sites with a high proportion of women, such as department stores, supermarkets, and call centers. A smoking cessation environment was created at business sites by placing women-targeted posters, leaflets, and banners on company bulletin boards and groupware. In-house smoking cessation campaigns were conducted to advertise smoking cessation services and to recruit participants. Most of those registered in the workplace were classified as emotional labor workers for service and sales. Furthermore, smoking cessation counseling was provided in collaboration with obstetrics and gynecological clinics and hospitals for pregnant women for whom smoking cessation was medically necessary.

2. Counseling methods

Individual counseling sessions were conducted based on the principle of confidentiality at institutions with secured, independent counseling rooms not exposed to the outside. For occupations with three shifts, counseling was operated flexibly, providing both day and night sessions. Further, smoking cessation and prevention education were provided at the workplace and at women’s welfare facilities. In accordance with the Privacy Act, after participants provided informed consent and understood the general parameters of the study, they received a preliminary consultation. After that, they signed up for smoking cessation counseling. Trained smoking cessation specialists who had majored in health science and counseling and completed national training for smoking cessation specialists conducted the smoking cessation counseling over six months. Moreover, telephone counseling was used when face-to-face counseling was difficult. The counseling session included strengthening smoking cessation motivation, controlling withdrawal symptoms and the desire to smoke, refusing to smoke, drinking management, and stress management. Face-to-face counseling involved a mandatory carbon monoxide measurement, and participants were provided with nicotine supplements (i.e., nicotine patches, lozenges, or chews) and various behavior-enhancing items (i.e., tooth-brushing set, mouthwash, mint candies, and grip strength systems) [29]. Considering the amount of smoking, level of nicotine addiction, and abstinence from supplements, nicotine supplements were provided within 12 weeks per person per year and up to 3 weeks per person. Additionally, health measurement services customized for women were provided, including stress tests, skin oils and moisture measurements, and an in-body check-up.

Re-smoking was considered a failure to quit smoking and participation in the study was terminated. If there was a loss of contact, it would be classified as a failure to quit smoking, since it could not be known whether the participant was smoking or not. To check whether an individual succeeds in smoking cessation, in-person checks must be done to determine whether or not the individual quit smoking such as through a carbon monoxide or cotinine test. If the individual passes the test, successful smoking cessation is declared.

3. Stress management

Smoking cessation counseling was conducted using the materials produced specifically including a quit-smoking diary, coloring book, and smoking cessation kit (i.e., tooth-brushing set, mouthwash, mint candies, and grip strength systems). Particularly, coloring books are proven as effective smoking cessation tools for relieving stress, providing emotional stability, and aiding participants in fighting cravings [30].

Emotional labor workers participated in the smoking cessation intervention program along with stress management, while workers of other occupations were provided basic smoking cessation counseling. Emotional labor workers for service and sales were more exposed to stress than other occupations due to the nature of their work, so the stress management part of the counseling was strengthened. As a method of psychological healing for emotional labor workers for service and sales, this study also provided in-depth stress tests and color therapy. The in-depth stress test involved measuring pulse waves using a heart rate variability measurement device [31] and the Perceived Stress Scale [32] to measure stress. Participants with a high level of stress were further examined using the depression scale [33]. Stress management counseling using color therapy has been reported to relieve stress and increase the vitality of life using the energy and properties of colors [34].

Those with low peer support tend to smoke much more than those with high peer support, and smokers are heavily influenced by the people who support them [35]. Emotional labor workers for service and sales were able to use peer support with their co-workers in the workplace. Peer support was provided as part of strengthening the motivation to quit smoking and managing stress. We conducted a buddy program to raise peer support to assist effective smoking cession. The participants were invited to select a nonsmoking individual from their social or familial groups who supported them to quit smoking and would help them to quit. They were provided with a program that motivated them to quit smoking together with work colleagues who were smokers. The buddy program was utilized to strengthen incentives to quit smoking. When cessation was maintained, behavioral enhancement materials were provided to cessation supporters and work colleagues who were smokers to help participants to continue cessation. Once they had successfully ceased smoking for six months, participants were provided with an award.

### 2.3. Statistical Analyses

Data analyses were performed with SPSS 24.0. *T*-tests and chi-square tests were performed to compare the emotional labor workers for service and sales and other occupations groups in terms of their general and smoking-related characteristics. A survival analysis using the Kaplan–Meier survival curves was conducted to determine how censored data (failure to follow up) affected emotional labor workers for service and sales and other occupations during the 24 week cessation period. Factors associated with success in smoking cessation depending on time points after intervention were analyzed using multivariable logistic regression by modifying the age, BMI, and drinking status.

## 3. Results

The average BMI of the emotional labor workers for service and sales was 22.02. A total of 26.1% of the participants comprised the high-risk drinking group, and 50.8% had started smoking before the age of 19 years. This percentage was significantly higher compared to the other occupations group. A total of 56.4% of the emotional labor workers for the service and sales group smoked 10 cigarettes or more per day. Heavy smokers, defined as having a carbon monoxide level of 21 ppm or higher [36], comprised 9.1% of the group, and 8.0% were classified as “high” in nicotine dependence, which was significantly higher than among other occupations. The Cramer’s V effect size value had the greatest correlation in alcohol drinking status (0.405) (Table 1).

In terms of motivation rulers, emotional labor workers for service and sales had average Importance, Confidence, and Readiness scores of 7.09, 5.18, and of 5.24, respectively, which were significantly lower than the averages of the other occupations. The average number of smoking cessation counseling sessions for the emotional labor workers for service and sales was 3.04, which was significantly higher than 1.84 sessions for the other occupations. The success rate of smoking cessation for the emotional labor workers for service and sales was 18.3% for 4 weeks, 16.6% for 6 weeks, 12.7% for 12 weeks, and 10.2% for 24 weeks, which was significantly higher for all periods compared to the other occupations (Table 2).

**Figure 1:**
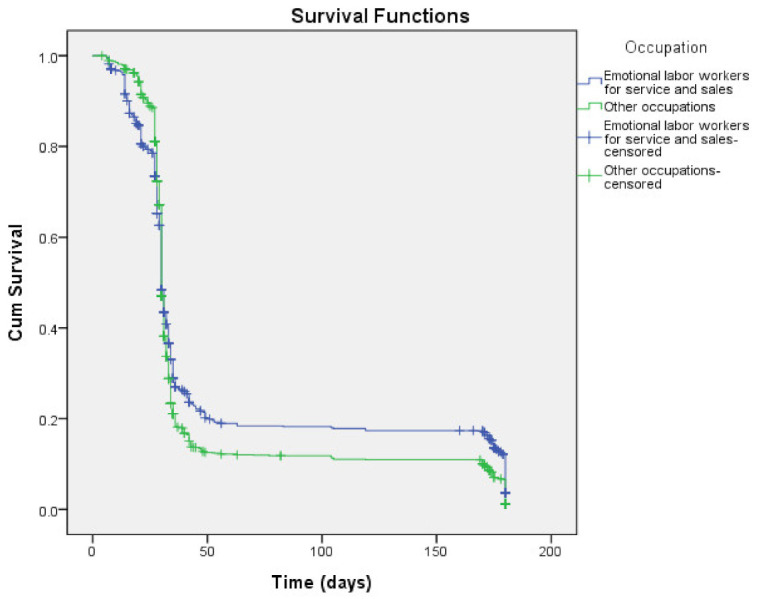
Comparison of Kaplan–Meier survival curves according to occupation (*p* = 0.003).

It was observed that emotional labor workers for the service and sales force quit smoking for longer. There was a statistically significant difference in women’s smoking cessation patterns depending on whether they were emotional labor workers in the service and sales group or not (*p* = 0.009; Figure 1).

The frequency of smoking cessation supporters was in the order of spouse/lover, work colleague, parents/grandparents, friends/school seniors and juniors, brothers and sisters, other, and siblings as emotional labor workers for service and sales. For other occupations, the order was spouse/lover, friends/school seniors and juniors, parents/grandparents, other, brothers and sisters, work colleagues, and siblings (Table 3).

To the question, “When is the most difficult time to resist the urge to smoke during the day?”, emotional labor workers for service and sales responded with the following in descending order: negative situations, habitual situations, in the morning right after waking up, during breaks, being with a smoker or seeing an actor/actress smoke on TV, before going to sleep, in the washroom/after shower, and positive situations. Other occupations responded with the following in descending order: in the morning right after waking up, habitual situations, and negative situations; the other options were ranked the same as emotional labor workers for service and sales (Table 4).

In terms of emotional labor status, emotional labor workers for service and sales were 2.27 times more likely to be successful in maintaining their nonsmoking status at 24 weeks compared to those in the other occupations. Participants were more likely to be successful in quitting smoking with a higher number of smoking cessation counseling sessions, and they were 5.35 times more likely to successfully maintain nonsmoking status at 24 weeks. Higher scores on motivation rulers (Importance, Confidence, and Readiness) were associated with longer periods of smoking cessation. The age at first smoking experience was significantly associated with smoking cessation at 6 and 24 weeks and smoking for the first time at an older age was associated with a higher likelihood of quitting. A lower daily average number of cigarettes smoked was associated with longer durations of smoking cessation. Lower levels of expired carbon monoxide and nicotine dependence were associated with longer durations of smoking cessation (Table 5).

## 4. Discussion

In this study, emotional labor workers for service and sales were more likely to be successful in quitting smoking compared to other occupations. Emotional labor workers in Korea comprise an occupational group with a high proportion of women and non-regular workers, who experience employment instability, low income, and long hours [1]. Consumers utilize their comparatively higher status, described in the phrase—“Customer is king” [37]—to make unreasonable demands on emotional labor workers, who provide service for these often-negative consumers [38]. This study focused on the differences in smoking-related variables between female emotional labor workers and other workers and variables related to abstinence success.

Low employment stability and high anxiety have a negative influence on health [39]. Non-regular work not only reduces job satisfaction but also raises health risks associated with drinking and smoking [40]. Nicotine addiction, pleasure from smoking, and resisting social pressure against quitting smoking, which all influence smoking behavior, are reported to be significantly higher among those with lower socioeconomic status [41]. As such, female emotional labor workers experience a reduction in their social interactions due to the fact of irregular working hours. In a job environment with little downtime and the need for quick rest, smoking becomes the only hobby that smokers are able to enjoy alone [42]. Cigarette breaks in a job environment where breaks are not guaranteed is a means to “squeeze in” a break. Existing studies indicate that women smokers have a stronger tendency to smoke to manage their stress compared to male smokers [43]. Female emotional labor workers require a means to vent emotionally after listening to and acting on complaints by consumers to be able to deal with a new consumer with a fresh mindset; they engage in smoking as a stress management behavior. However, in the present study, we observed that success rates of smoking cessation for women emotional labor workers for service and sales at the 4, 6, 12, and 24 week periods were all higher than those for other occupations, along with a higher average number of smoking cessation counseling sessions. This indicates that even though female emotional labor workers for service and sales were more addicted to nicotine and faced more difficulties in quitting smoking than other occupations, high-intensity smoking cessation counseling with stress management increased the probability of successful smoking cessation. Existing studies indicate that women experience more chronic stress, as well as daily stress, and have more psychological concerns compared to men [44,45]. Further, smokers reported lower stress scores compared to nonsmokers [46]. Therefore, we conducted high-intensity counseling for stress management with color therapy and stress testing in female emotional labor workers for service and sales. Emotional labor workers, on average, had higher daily numbers of cigarettes smoked, CO levels, and nicotine dependence than other occupations. However, as a result of conducting an intervention with stress management, the success rate of smoking cessation was higher for 6 months— clinically, a very meaningful result.

Social support is a key factor that influences health behaviors, such as smoking and drinking, and this association is approximately twice as strong among women compared to men [47]. Since women face more difficulties in quitting smoking if social support is lacking, more social support is needed for women smokers to attempt to quit. In this study, women emotional labor workers for service and sales indicated that work colleagues were second-most helpful supporters next to the spouse/lover. To enable effective quitting, we employed a buddy program in which work colleagues became cessation supporters at work, where participants spent most of their time. We also incorporated the group quitting method, where smokers in the high-risk smoking group who were determined to quit became supporters for each other as well as smokers and nonsmokers were placed into a support group as a team to encourage quitting. While both methods were effective, teams composed of only smokers had the possibility of failing to quit if any member smoked; nevertheless, they were effective in deciding to quit smoking. However, the group of female emotional labor workers for service and sales who received stress management, smoking cessation counseling, and peer support had a higher likelihood of quitting at all time points assessed, compared to other occupations who were not subject to this method. This indicates that high-intensity smoking cessation counseling, including stress management and social support, had a significant influence on smoking cessation among female emotional labor workers for service and sales.

When developing the guidelines on smoking cessation, the US Centers for Disease Control and Prevention conducted a meta-analysis to identify the effects of smoking cessation interventions, which were effective when associated with longer counseling periods and a higher number of sessions [48]. We also observed an increased likelihood of successful quitting with higher numbers of smoking cessation counseling sessions. This appears to be because the sessions involved checking the status of the participant’s smoking cessation and providing appropriate information related to smoking cessation and nicotine replacement therapies. Stress management was instrumental in strengthening the smoking cessation behavior of women smokers.

In a study of women college students who were smokers, among the motivation rulers, Confidence and Readiness were significant factors for smoking cessation, whereas Importance was not significant in terms of successful quitting [29]. Contrary to existing research, this study found all motivation rulers, that is, Importance, Confidence, and Readiness, to be significant, with higher scores being associated with longer periods of smoking cessation. The measurement of motivational rulers at baseline among teenagers has been associated with predicting the daily average number of cigarettes smoked in a 12 month follow-up [49]. Other evidence suggests that motivational rulers are significant predictors of changes in smoking behavior [28]. In this study, women emotional labor workers for service and sales had lower average scores for the three elements of motivational rulers compared to other occupations. This led to the prediction that women emotional labor workers would have smoked a higher number of cigarettes on a daily basis, as well as have had a lower chance of success in smoking cessation.

Our results revealed that the age at first smoking experience was significantly associated with 6 and 24 week periods of smoking cessation. A previous study indicated that the earlier age of the first cigarette led to more cigarettes smoked due to the fact of nicotine addiction [50]. For successful smoking cessation, it is important to delay the age at which the first cigarette is smoked. Further research is needed to examine the factors associated with the first experience of smoking among women.

Our results also indicated that lower expired carbon monoxide levels were associated with longer periods of smoking cessation. Tests for expired carbon monoxide examine the residual carbon monoxide in expired air from the participants, identifying smokers and nonsmokers and smoking habits [51]. Given that expired carbon monoxide is strongly related to success in smoking cessation, it is important to measure expired carbon monoxide levels in each counseling session, and to communicate the importance of lowering the levels of expired carbon monoxide and enhance the motivation to quit smoking.

We observed that a lower number of cigarettes smoked in a day and lower nicotine dependence were associated with longer durations of smoking cessation. These results are consistent with existing studies indicating that a lower number of cigarettes smoked in a day is associated with successful smoking cessation [52,53]. As women react more sensitively to nicotine compared to men, nicotine metabolizes slower in women, remaining in their body for longer periods than for men [54]. Women tend to depend on nicotine on a psychological basis. Since female emotional labor workers for service and sales smoke more cigarettes and have higher nicotine dependence compared to other occupations, it is necessary to provide this population with higher-intensity smoking cessation counseling.

Successful smoking cessation among women might be less likely than among men [55], and existing studies indicate that continued smoking cessation over the long term is less likely for women [56]. For this study, we developed a 24 week smoking cessation program, and our results indicate the need for a customized counseling program for women smokers to assist them in refraining from smoking in the long term, for periods of more than a year.

There were several limitations to this study. First, we examined adult women smokers in certain regions of Seoul and conducted counseling for women emotional labor workers for service and sales in which the proportion of smokers was higher. This limits the generalization of the results of this study, and repeated follow-up research should examine adult women in various regions and occupations.

The second limitation was that the number of variables was limited because this study used secondary data, and the association of various variables (economic characteristics, average monthly income, parental education level, health awareness, stress level, etc.) and non-smoking success was unknown. Furthermore, we could not control the potential confounder that was not observed.

Third, although compliance with medication usage was not included in this study, counselors asked subjects about nicotine supplements and recorded them at each counseling session.

Fourth, there were no follow-up data on the participants whose data were discontinued due to smoking again, leaving the organization, or loss of contact information, which might have resulted in a bias.

One of the reasons for the high dropout rate was the registration in anti-smoking counseling in health-related campaigns to promote smoking cessation. These mostly included women in other occupations. Health-related campaigns have been held several times on a one-off basis in various places, including squares, streets, and playgrounds, where many people gather. It was difficult to manage later because the consultation did not start at the same place and at a set time. Another reason was that emotional labor workers for service and sales left the company and frequently moved because most of them were non-regular and temporary workers. As most women are reluctant to let people around them know that they are smokers, women who came for smoking cessation counseling told counselors that the fact that they were receiving smoking cessation counseling would inform others that they smoke. The participation and success rate for visiting smoking cessation counseling were expected to be higher since it has relieved subjects from their burden of visiting public health center clinics for smoking cessation themselves. Nevertheless, subjects felt burdened to participate in counseling at the workplace. Thus, we need a specialized anti-smoking counseling program for women. The 24 week success rate may be quite low, but it is a very important and meaningful achievement that led to the smoking cessation of women, which has been a blind spot until now.

This is the first study to employ data from a six-month smoking cessation counseling program provided by a smoking cessation counselor to female smokers who are emotional labor workers for service and sales. Our findings are novel and valuable, given that extant research has focused on smoking among adult men and adolescents and has neglected smoking behaviors and cessation patterns among adult women.

## 5. Conclusions

This study was conducted to identify the factors associated with successful smoking cessation through tailored smoking cession counseling with stress management among female emotional labor workers for service and sales. The results indicated that the factors associated with the duration of smoking cessation were the number of counseling sessions, motivation rulers (Importance, Confidence, Readiness), average daily number of cigarettes smoked, expired carbon monoxide, and nicotine dependence; the most significantly associated factor was the number of counseling sessions.

## Figures and Tables

**Table 1:** Health and smoking-related characteristics according to labor status.

Variable		Emotional Labor Workers for Service and Sales *N* = 1002 (%)	Other Occupations *N* = 1672 (%)	Total *N* = 2674 (%)	*p*-Value	Effect Size ^a^
Age	Mean ± SD	30.42 ± 9.03	30.42 ± 10.07	30.42 ± 9.69	0.986	0.181
BMI	Mean ± SD	22.02 ± 4.33	20.56 ± 3.46	21.55 ± 4.13	<0.001	0.207
Alcohol drinking status						0.405
High-risk drinking *		262 (26.1)	120 (7.2)	382 (14.3)	<0.001	
Moderate drinking		266 (26.5)	127 (7.6)	393 (14.7)		
No		474 (47.3)	1425 (85.2)	1899 (71.0)		
Age of first smoking experience (years)	Mean ± SD	19.49 ± 5.54	20.14 ± 6.05	19.89 ± 5.87	0.006	0.182
≤15		184 (18.4)	241 (14.4)	425 (15.9)	<0.001	
16–18		325 (32.4)	452 (27.0)	777 (29.1)		
≥19		493 (49.2)	979 (58.6)	1472 (55.0)		
Cigarettes per day	Mean ± SD	9.66 ± 5.51	9.03 ± 6.48	9.26 ± 6.14	0.008	0.172
<5		145 (14.5)	369 (22.1)	514 (19.2)	<0.001	
5–9		292 (29.1)	469 (28.1)	761 (28.5)		
10–14		346 (34.5)	539 (32.2)	885 (33.1)		
≥15		219 (21.9)	295 (17.6)	514 (19.2)		
CO level (ppm)	Mean ± SD	10.71 ± 7.14	7.43 ± 6.46	8.75 ± 6.93	<0.001	0.232
0–6		300 (31.3)	758 (53.1)	1058 (44.3)	<0.001	
7–10		243 (25.3)	321 (22.5)	564 (23.6)		
11–20		330 (34.4)	282 (19.7)	612 (25.6)		
≥21		87 (9.1)	67 (4.7)	154 (6.4)		
Nicotine dependency	Mean ± SD	2.79 ± 2.37	2.50 ± 2.29	2.61 ± 2.32	0.002	0.087
Low (0–3)		625 (62.4)	1148 (68.7)	1773 (66.3)	0.003	
Mild (4–6)		297 (29.6)	424 (25.4)	721 (27.0)		
High (≥7)		80 (8.0)	100 (6.0)	180 (6.7)		
Experience of quitting smoking						0.006
Yes		464 (46.3)	785 (46.9)	1249 (46.7)	0.747	
No		538 (53.7)	887 (53.1)	1425 (53.3)		

* High-risk drinking group: more than five drinks a day (or three cans of beer) twice or more per week. ^a^ Effect size Cramer’s V: 0.1 = weak relationship, 0.3 = moderate relationship, 0.5 = strong relationship.

**Table 2:** Success factors in quitting smoking according to labor status.

Variable		Emotional Labor Workers for Service and Sales *N* = 1002 (%)	Other Occupations *N* = 1672 (%)	Total *N* = 2674 (%)	*p*-Value	Effect Size ^a^
Motivation rulers						
Importance	Mean ± SD	7.09 ± 2.40	7.11 ± 2.37	7.10 ± 2.38	<0.001	0.056
Confidence	Mean ± SD	5.18 ± 2.35	5.77 ± 2.58	5.55 ± 2.51	<0.001	0.141
Readiness	Mean ± SD	5.26 ± 2.44	5.84 ± 2.59	5.62 ± 2.55	<0.001	0.132
Frequency of counseling	Mean ± SD	3.04 ± 2.74	1.84 ± 1.66	2.28 ± 2.20	<0.001	0.195
1		318 (33.3)	1020 (61.3)	1338 (51.1)	<0.001	
2		263 (27.5)	367 (22.0)	630 (24.0)		
3		134 (14.0)	137 (8.2)	271 (10.3)		
4		64 (6.7)	41 (2.5)	105 (4.0)		
≥5		176 (18.4)	100 (6.0)	276 (10.5)		
Smoking abstinence						
4 weeks		183 (18.3)	171 (10.2)	354 (13.2)	<0.001	0.115
6 weeks		166 (16.6)	157 (9.4)	323 (12.1)	<0.001	0.107
12 weeks		127 (12.7)	107 (6.4)	234 (8.8)	<0.001	0.107
24 weeks		102 (10.2)	63 (3.8)	165 (6.2)	<0.001	0.129

^a^ Effect size Cramer’s V: 0.1 = weak relationship, 0.3 = moderate relationship, 0.5 = strong relationship.

**Table 3:** Supporters of quitting smoking (multiple responses).

Variable	Emotional Labor Worker for Service and Sales		Other Occupations	
	1002 (%)	Rank	1672 (%)	Rank
Spouse/lover	241 (24.1)	1	400 (23.9)	1
Work colleagues	220 (22.0)	2	107 (6.4)	6
Parents/grandparents	144 (14.4)	3	313 (18.7)	3
Friends/school seniors and juniors	137 (13.7)	4	325 (19.4)	2
Brothers and sisters	60 (6.0)	5	124 (7.4)	5
Others	58 (5.8)	6	147 (8.8)	4
Sons and daughters	37 (3.7)	7	59 (3.5)	7

**Table 4:** When is the most difficult time to resist the urge to smoke during the day? (multiple responses).

Variable	Emotional Labor Worker for Service and Sales		Other Occupations	
	1002 (%)	Rank	1672 (%)	Rank
After meals	416 (41.5)	1	634 (37.9)	1
Negative situations (when stressed, when things did not go as expected, when you get angry, etc.)	357 (35.6)	2	418 (25.0)	3
Habitual situations (when you need refreshing/when you realize you had not smoke for a while/when you are drinking alcohol or coffee/when you are alone or waiting, etc.)	315 (31.4)	3	448 (26.8)	2
In the morning right after waking up	219 (21.9)	4	322 (19.3)	4
During breaks	115 (11.5)	5	175 (10.5)	5
Being with a smoker or seeing an actor/actress smoke on TV	104 (10.4)	6	159 (9.5)	6
Before going to sleep	79 (7.9)	7	105 (6.3)	7
In the washroom/after shower	59 (5.9)	8	101 (6.0)	8
Etc.	28 (2.8)	9	48 (2.9)	9
Positive situations (when you are with friends or family/when you are chattering or relaxing, etc.)	19 (1.9)	10	47 (2.8)	10

**Table 5:** The factors associated with successfully of quitting smoking.

Variable	4 Weeks (*n* = 354)	6 Weeks (*n* = 323)	12 Weeks (*n* = 234)	24 Weeks (*n* = 165)
	Odds Ratio (95% Confidence Interval)	Odds Ratio (95% Confidence Interval)	Odds Ratio (95% Confidence Interval)	Odds Ratio (95% Confidence Interval)
Emotional labor status	1.33 (0.96–1.85)	1.23 (0.88–1.71)	1.26 (0.87–1.83)	2.27 (1.39–3.72) **
Frequency of counseling	4.54 (3.82–5.40) ***	4.36 (3.66–5.18) ***	4.75 (3.85–5.87) ***	5.35 (4.03–7.10) ***
Motivation rulers				
Importance	1.21 (1.13–1.30) ***	1.23 (1.15–1.32) ***	1.29 (1.18–1.40) ***	1.24 (1.13–1.36) ***
Confidence	1.24 (1.17–1.32) ***	1.24 (1.16–1.32) ***	1.31 (1.22–1.40) ***	1.31 (1.21–1.42) ***
Readiness	1.22 (1.15–1.30) ***	1.22 (1.14–1.29) ***	1.31 (1.22–1.40) ***	1.25 (1.15–1.35) ***
Age of first smoking experience (years)	1.03 (1.00–1.06)	1.04 (1.01–1.07) *	1.02 (0.99–1.06)	1.04 (1.00–1.08) *
Cigarettes per day	0.93 (0.90–0.95) ***	0.93 (0.90–0.96) ***	0.94 (0.91–0.97) ***	0.90 (0.87–0.94) ***
CO level (ppm)	0.50 (0.42–0.60) ***	0.47 (0.39–0.57) ***	0.40 (0.32–0.51) ***	0.39 (0.30–0.51) ***
Nicotine dependency	0.86 (0.80–0.91) ***	0.64 (0.49–0.82) **	0.63 (0.47–0.85) **	0.46 (0.31–0.67) ***

* *p* < 0.05, ** *p* < 0.01, *** *p* < 0.001, adjusted by age, BMI, drinking.

## Data Availability

The data supporting the findings of this study are available only to researchers who participated in 17 Tobacco Control Centers in Korea. The data are not publicly available due to privacy.
